# Assessment of Four Essential Features of the *Alive & Thrive* Initiative to Improve Maternal, Infant, Young Child, and Adolescent Nutrition

**DOI:** 10.1111/mcn.13800

**Published:** 2025-01-27

**Authors:** Edward A. Frongillo, Jessica Escobar‐DeMarco, Sujata Bose

**Affiliations:** ^1^ Department of Health Promotion Education, and Behavior, University of South Carolina Columbia South Carolina USA; ^2^ Department of Epidemiology and Community Health University of North Carolina at Charlotte Charlotte North Carolina USA; ^3^ ELEVATE Nutrition Washington District of Columbia USA

**Keywords:** advocacy, behaviour change, policy, quality assurance, quality improvement, technical assistance

## Abstract

*Alive & Thrive* has been a major global nutrition initiative that aimed to learn how to improve maternal, infant, young child, and adolescent nutrition and health on a large scale. During 2009–2014, *Alive & Thrive* developed and implemented interventions to improve infant and young child feeding at scale in three countries. Subsequently, *Alive & Thrive* expanded its work to more than 15 geographies, including six country‐specific and two regional programs, to additionally address maternal and adolescent nutrition while adding agriculture and social protection programs to improve maternal, infant, and young child nutrition. Reflecting on the big picture of what the *Alive & Thrive* initiative has accomplished in its 16 years and how it did so, four features warranted emphasis because additional learning was needed. These four features were: commitment to exemplary large‐scale social and behaviour change communication, explicit focus on policy and advocacy to achieve and sustain change, promotion of country‐led initiatives through technical assistance, and attention to quality assurance and improvement in programming. For each feature, an assessment across the *Alive & Thrive* portfolio was commissioned, each led by an external consultant or team. The assessments highlighted designing and implementing exemplary social and behaviour change programming and policy advocacy in each country, undertaking a large‐scale effort through careful, in‐depth, and evidence‐based planning that is bolstered by strong engagement with relevant stakeholders at multiple levels. The assessments also highlighted technical assistance that is responsive to country contexts to support country‐led initiatives and attention to assuring and improving the quality of programming to achieve effectiveness.

## Summary

- *Alive & Thrive* worked in more than 15 geographies to develop and implement interventions to improve infant and young child, maternal, and adolescent feeding and nutrition while adding agriculture and social protection programs.
- Four features of *Alive & Thrive* warranted emphasis; for each an assessment across the portfolio was led by an external consultant or team.
- The assessments highlighted designing and implementing exemplary social and behaviour change programming and policy advocacy; careful, in‐depth, and evidence‐based planning engaging with stakeholders at multiple levels; technical assistance responsive to country initiatives; and assuring and improving quality of programming.

## Graphical abstract

*Alive & Thrive* has learned how to improve maternal, child, and adolescent nutrition and health through commitment to large‐scale social and behaviour change communication, focus on policy and advocacy to achieve and sustain change, promotion of country‐led initiatives through technical assistance, and attention to quality assurance and improvement in programming.

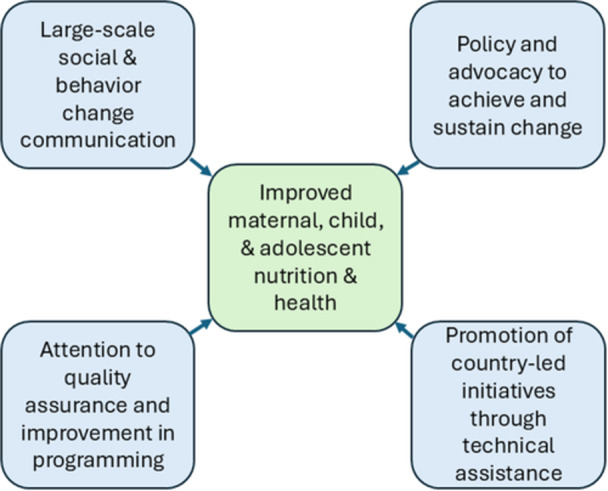

## 1 Introduction

For 16 years, *Alive & Thrive*, funded by the Bill & Melinda Gates Foundation and other contributors, has been a major global nutrition initiative to learn how to improve maternal, infant, young child, and adolescent nutrition and health on a large scale. During its first generation from 2009 to 2014, *Alive & Thrive* developed and implemented interventions aimed at improving infant and young child feeding at scale in Ethiopia, Bangladesh, and Vietnam. Subsequently, in a second generation, *Alive & Thrive* expanded its work to more than 15 geographies to additionally address maternal and adolescent nutrition while adding agriculture and social protection activities to improve maternal, infant, and young child nutrition. These geographies included country‐specific programs in Bangladesh, Burkina Faso, Ethiopia, India, Madagascar, and Nigeria and regional programs in East Asia Pacific and West Africa. *Alive & Thrive* has used its extensive network, expertize, and experience to work with governments, United Nations agencies, non‐governmental organizations, universities, and others to provide strategic technical assistance and conduct focused research to help strengthen systems, build capacity, and support sustainability in these and other countries in Africa and Asia and to share innovative interventions, methods and tools, and lessons learned. Furthermore, *Alive & Thrive* focused its 2017–2022 implementation research on testing the integration of nutrition services into different platforms (e.g., maternal nutrition into antenatal care, urban settings, private sector, the education sector, polio immunization platform), assessing the effectiveness of policies that support optimal infant feeding, and learning about adaptations needed to continue essential services during crises, generating knowledge on how to improve nutrition practices while safeguarding essential health and nutrition services for women and children.

*Alive & Thrive* has been one of the six initiatives that aim to improve maternal, infant, and young child nutrition on a large scale. Three other initiatives each focused on one country: the *National Food Nutrition Programme* in Ecuador (Lutter et al. 2008), *Suaahara* in Nepal (Frongillo et al. 2024), and *Estrategia Integral de Atención a la Nutrición* in Mexico (Frongillo 2020). The national programme in Ecuador provided a fortified complementary food starting at 6 months of age. The programme in Nepal provided integrated programming to improve maternal and young child nutrition across multiple sectors (i.e., nutrition, health, family planning, water, sanitation, and hygiene) in more than half of Nepal's districts. The national programme in Mexico aimed to strengthen the health and nutritional component of the country's conditional cash transfer programme to address the nutritional transition and improve the health and nutrition of beneficiaries. Two other initiatives worked in multiple countries: *LINKAGES* during 1996–2006 (United States Agency for International Development 2006) and *Assessment & Research on Child Feeding* during 2012–2023 (Helen Keller International. Assessment & Research on Child Feeding Project. 2023). Impact evaluations of *Alive & Thrive* in three countries (Menon et al. 2016a, 2016b; Rawat et al. 2017; Kim et al. 2018; Kim et al. 2019), the *National Food Nutrition Programme* in Ecuador (Lutter et al. 2008), and *Suaahara* in Nepal (Frongillo et al. 2024) have demonstrated effectiveness in improving maternal, infant, and young child nutrition on a large scale. *Alive & Thrive* stands out for undertaking comparative efforts in three countries initially and then using lessons learned to replicate, adapt, promote, and support interventions in multiple other countries.

From its inception, *Alive & Thrive* drew on ideas from multiple behavioural and community models and built its work at four socio‐ecological levels (i.e., individual, interpersonal, community, and enabling environment) through research, design, and planning, partnerships and alliances, and engagement with policymakers (Frongillo 2020; Sanghvi et al. 2022). *Alive & Thrive* conducted in‐depth investigations using mixed methods in each country in which it worked. It developed tailored strategies, theories of change and impact paths, context‐specific implementation guides, standard operation procedures with monitoring tools, and plans for effective implementation and adoption. *Alive & Thrive* worked through partnerships and alliances to design and implement interventions that included advocacy at national and subnational levels, interpersonal communication, mass and local media, and the strategic use of data. The interventions used global nutrition recommendations for maternal, infant, and young child nutrition and were implemented through large‐scale platforms to maximize scalability. *Alive & Thrive* engaged with policymakers and staff at multiple levels and sectors, as well as service providers, community leaders, families, mothers, and caregivers.

Reflecting on the big picture of what the *Alive & Thrive* initiative has accomplished in its 16 years across multiple geographies and how it did so, four features stood out as warranting emphasis because additional learning was needed regarding implementation. These four features were as follows: commitment to exemplary large‐scale social and behaviour change communication, explicit focus on policy and advocacy to achieve and sustain change, promotion of country‐led initiatives through technical assistance, and attention to quality assurance and improvement in programming. These four features were identified through an iterative process involving the *Alive & Thrive* Strategic Technical Advisory Group and staff from the Bill & Melinda Gates Foundation and *Alive & Thrive*. An assessment across the *Alive & Thrive* portfolio for each of these four features was commissioned, each led by an external consultant or team, to surface and promote lessons learned about how to do each. These assessments resulted in four articles in *Maternal & Child Nutrition* (Flax et al. 2023; Anderson et al. 2024; Siekmans et al. 2024; Sethuranam et al. 2024).

### 1.1 Social and Behaviour Change Communication

Social and behaviour change communication was at the core of the programming of *Alive & Thrive*. Flax et al. (2023) reviewed programme documents and peer‐reviewed articles to describe the implementation processes used by *Alive & Thrive* in its social and behaviour change programming and conducted key informant interviews with 23 *Alive & Thrive* staff members and stakeholders in six countries: Bangladesh, Burkina Faso, Ethiopia, India, Nigeria, and Vietnam. *Alive & Thrive* used interpersonal communication, community mobilization, and mass media through multiple channels and frequent contacts to improve knowledge, self‐efficacy, and social norms, resulting in increased exclusive breastfeeding, minimum meal frequency of children, and uptake of iron and folic acid by pregnant women, with mixed effects on early initiation of breastfeeding and maternal and child dietary diversity. Essential to achieving impact were using available large‐scale platforms for interpersonal communication, enhancing counselling skills of health workers, providing messages that were timely and tailored, encouraging important influencers to take specific actions, using research to address behavioural concerns, and increasing reach and frequency of mass media, use of simple and memorable messages, and adding channels to reach those with low media coverage. These intervention strategies can be adopted and used by governments and other implementors in countries wishing to improve maternal, infant, and young child nutrition.

### 1.2 Policy and Advocacy

Maternity protection policies that preserve the health of working women and their infants promote infant and young child nutrition through breastfeeding, and prevent workplace discrimination against women are important and cost‐effective, but more knowledge is needed on how advocates can effectively champion maternity protection policies and their implementation in different country contexts (Anderson et al. 2024). Anderson et al. assessed advocacy efforts by *Alive & Thrive* in Indonesia, Nigeria, the Philippines, and Vietnam to understand how advocates may effectively advance maternity protection policies for maternal leave and workplace lactation. A desk review of programme and policy documents was conducted, and a total of 20 interviews with 17 diverse stakeholders were interviewed. The assessment identified several principles and actions that contributed to effective advocacy for maternity protection policies. For example, effective advocacy efforts used partnerships with traditional and non‐traditional stakeholders that were informed by opinion leader research and strengthened through consensus‐building. Consideration of country context helped to identify attainable policy requests and develop advocacy strategy; this included attention to the governance structure in the country, the country's ability to fund social protections, and current government priorities to determine the best possible angle for advocacy (e.g., health vs*.* economic benefits). Findings also suggest the usefulness of presenting support for maternity leave and workplace lactation as social protections that have synergistic benefits. These principles and actions can help policy champions design and implement advocacy for maternity protection policies in other countries.

### 1.3 Technical Assistance

In the second generation of *Alive & Thrive* when expanding its work to multiple geographies, the initiative provided strategic technical assistance to countries to help them develop effective policies; improve the design and implementation of maternal, infant, and young child nutrition programs; and strengthen the capacity of systems to deliver and sustain programming at scale and with quality. Siekmans et al. (2024) assessed the provision of technical assistance by conducting a document review and 79 stakeholder interviews, with the aim to describe the technical assistance provided in six countries, assess how the contextual and technical assistance processes affected the results achieved, and document lessons learned. The assessment, to be manageable, focused on technical assistance both for strengthening national policy formulation, monitoring, and implementation of the International Code of Marketing of Breast‐Milk Substitutes and for supporting the scale‐up of maternal nutrition services and strategic use of data. These foci were selected after an extensive review of the entire portfolio to be representative of the technical assistance done by *Alive & Thrive* and allow comparability since these activities were undertaken in more than one setting. Identifying and engaging with influential stakeholders who were not traditionally included (e.g., professional medical associations), using evidence for advocacy and decision‐making, using a diversity of methods to strengthen capacity, developing sets of tools for scaling up programs, and reinforcing means for feedback to improve service provision and data quality were important for technical assistance to be effective. Political shifts, poor functioning of health systems, and constrained resources to replicate or sustain progress inhibited effectiveness. The assessment found that ongoing investment in technical assistance based on local and practical evidence will strengthen the incorporation of nutrition into institutions (e.g., government, medical associations, civil society, and development partners). Furthermore, to achieve scale‐up, technical assistance is needed to help governments strengthen system capacity for nutrition, including by addressing inadequate financial and human resources.

### 1.4 Quality Assurance and Improvement

Although nutrition programmers generally understand that, to be effective, programs and services must be implemented with adequate coverage, intensity, and quality, what the latter should mean for nutrition programs has not been developed well. Drawing on the work of *Alive & Thrive* in five countries (i.e., Bangladesh, Burkina Faso, Cambodia, Ethiopia, and India), Sethuranam et al. (2024) assessed which quality frameworks were used, which programme and programme components were addressed, and ways to improve quality of nutrition services for mothers, infants, young children, and adolescents. Programme documents were reviewed, and 30 key informant interviews were conducted with *Alive & Thrive* country staff members and stakeholders who were involved with service provision that was supported by *Alive & Thrive* technical assistance. The frameworks used by countries were either (or both) health‐system‐level quality assurance (i.e., largely systems strengthening) or facility‐level continuous quality improvement using an iterative process. That quality assurance or improvement was feasible was demonstrated by pilot interventions jointly supported by *Alive & Thrive* and country governments. Common quality assurance or improvement activities were improving the standards of care for nutrition, building skills of service providers, harmonizing training materials, mentoring and coaching staff, training supervisors and front‐line workers, providing supportive supervision, developing action plans to track progress, improving how services were delivered, altering counselling to emphasize dialogue rather than didactic teaching, promoting the strategic use of data to improve service provision, and strengthening the enabling environment. These activities can be adopted and used to enhance the effectiveness of future nutrition programming. The set of activities helps to advance understanding of what should constitute quality nutrition programming. Quality programming was facilitated by working in partnership with governments, aligning with government priorities, advocating for increased investment in quality assurance or improvement to enable implementation at scale, implementing quality activities at various levels within the health system, working simultaneously bottom‐up and top‐down, jointly implementing pilot interventions with governments, and scaling up when feasible.

## 2 Conclusions

The commitment and investment over 16 years to *Alive & Thrive* by many institutions, scientists, programmers, community staff, funders, and others to learn how to improve maternal, infant, and young child nutrition and health at large scale within and across countries, and then apply what has been learned, has been unprecedented in the field of nutritional sciences and remarkable for its long‐term vision. Funders of *Alive & Thrive* intended from the beginning that their investment of resources be both in the design and implementation of actions and research and evaluation about those actions. Furthermore, *Alive & Thrive* was designed to ensure that the actions and their impact would be sustained within countries (Kim et al. 2018) and through sharing and replicating what was learned with other countries. The four portfolio‐wide assessments summarized here have highlighted how to design and implement exemplary social and behaviour change programming and policy advocacy in each country undertaking a large‐scale effort to improve maternal, infant, and young child (and adolescent) nutrition through careful in‐depth and evidence‐based planning that is bolstered by strong engagement with relevant stakeholders at multiple levels. The assessments have also highlighted that technical assistance that is responsive to country contexts is needed to support country‐led initiatives and that attention to assuring and improving the quality of programming is crucial to achieving effectiveness. The four assessments identify ongoing challenges to implementation that can be addressed by governments and their partners in future programming and add a portfolio‐wide perspective on how to address implementation to an extensive and rich body of evidence that has been produced by *Alive & Thrive*.

## Author Contributions

Edward A. Frongillo conceptualized and developed the manuscript and is responsible for its content. Jessica I. Escobar‐Demarco and Sujata Bose reviewed and provided inputs on the manuscript. All authors read and approved the final version.

## Conflicts of Interest

The authors declare no conflicts of interest.

## Data Availability

The authors have nothing to report.
